# Metabolites of flavonoid compounds preserve indices of endothelial cell nitric oxide bioavailability under glucotoxic conditions

**DOI:** 10.1038/nutd.2017.34

**Published:** 2017-09-11

**Authors:** Y Qian, P V A Babu, J D Symons, T Jalili

**Affiliations:** 1 Department of Nutrition and Integrative Physiology, University of Utah, Salt Lake City, UT, USA; 2 Division of Endocrinology, Metabolism, and Diabetes, University of Utah School of Medicine, Salt Lake City, UT, USA; 3 Molecular Medicine Program, University of Utah, Salt Lake City, UT, USA

## Abstract

We hypothesized that metabolites of dietary flavonoids attenuate impairments in nitric oxide (NO) bioavailability evoked by glucotoxic conditions mimicking Type 1 or 2 diabetes. To test this, human aortic endothelial cells were treated with either vehicle control, quercetin-3-*O*-glucoronide, piceatannol or 3-(3-hydroxyphenyl)propionoic acid for 24 h. These are metabolites of quercetin, resveratrol and proanthocyanidin, respectively. Next, cells were exposed to control (5 mM) or high (25 mM) glucose conditions for 48 h, followed by insulin treatment (100 nM, 10 min) to stimulate NO production. In control glucose conditions NO production, phosphorylated to total endothelial nitric oxide synthase (p-eNOS^ser1177^: eNOS), and phosphorylated to total Akt (p-Akt^Ser473^: Akt) were all increased by insulin stimulation. This response was abolished during high glucose conditions. Pretreatment of cells with flavonoid metabolites prior to high glucose challenge preserved insulin stimulated increases in NO production, p-Akt^Ser473^: Akt and p-eNOS^Ser1177^: eNOS. These effects may be secondary to oxidative stress as pretreatment with all flavonoid metabolites prevented elevations in reactive oxygen and nitrogen species in response to high glucose. These data support the hypothesis that beneficial effects of flavonoids on endothelial cell function in the context of glucotoxicity, at least in part, are secondary to their metabolites.

## Introduction

Both flavonoid containing botanicals and isolated flavonoids can improve vascular function and blood pressure in animal models and humans. For example, grape seed extract lowers arterial blood pressure in subjects with metabolic syndrome,^1^ while resveratrol and quercetin improve endothelial function^2, 3^ and reduce blood pressure in hypertensive humans and animals.^4, 5, 6, 7^ We recently reported that a combination of quercetin, resveratrol, grape seed extract and green tea reduced blood pressure in hypertensive humans with metabolic syndrome.^8^ *In vitro* studies suggest such improvements are due to increased endothelial nitric oxide synthase (eNOS) phosphorylation leading to greater nitric oxide production.^9, 10^ To date, the majority of *in vitro* studies conducted to elucidate the responsible mechanisms have used the parent flavonoids, and not their metabolites. However, given the rapid metabolism of ingested flavonoids, and absence of parent flavonoids in human and animal plasma, the question of metabolite bioactivity arises. Therefore, the purpose of this study was to evaluate flavonoid metabolites in an *in vitro* model of hyperglycemia using cultured endothelial cells. While thousands of candidates exist, we selected a metabolite of quercetin, proanthocyanidin, and resveratrol, since each of these parent compounds, or botanicals containing them (for example, grape seed extract) have been studied and their efficacy demonstrated. Specifically, we examined: (i) quercetin-3-*O*-glucoronide (Q3G), a conjugated form of quercetin commonly found in human plasma; (ii) 3-(3-hydroxyphenyl)propionoic acid (3-HPP), a metabolite of proanthocyanidins which are prevalent in blueberries and grapes and (iii) piceatannol (PIC), a resveratrol metabolite. We hypothesized that these flavonoid metabolites prevent the suppression of insulin-stimulated nitric oxide (NO) production evoked by glucotoxicity. In support of this hypothesis, each metabolite rendered insulin-stimulated NO generation refractory to glucotoxicity, potentially by ameliorating oxidative stress.

## Methods

### Cell culture

Human aortic endothelial cells (HAEC) (Lonza, Carlsbad, CA, USA) were cultured in T-75 flasks using M199 Media (Gibco, CA, USA) containing 2% FBS and endothelial growth supplements (Life Technologies, CA, USA) in a humidified atmosphere (5% CO_2_/95% O_2_, 37 °C) as previously described.^8, 11^ HAEC were passaged at 80% confluence, used for experiments at 70–80% confluence during passages 4–6.

### Flavonoid metabolites

Quercetin-3-*O*-glucoronide and 3-HPP were purchased from Sigma Chemical (St Louis, Missouri, USA). Piceatannol was purchased from AG Scientific (San Diego, CA, USA).

### Cell treatments

HAEC were incubated overnight in serum free media. Cells pre-treated with either vehicle (DMSO), 1 μM 3-HPP, 5 μM PIC or 2 μM Q3G for 24 h, followed by 48 h incubation in low (5 mM) or high (25 mM) glucose. At the end of the treatment period, cells were treated with insulin (100 nM, 10 min) a known stimulator of eNOS phosphorylation and NO production.^12, 13^ HAEC treated with 5 mM glucose were also supplemented with 20 mM mannitol to control for osmolarity.^14^ Cell viability was assessed as previously described.^13^

### NO production

Stable nitrate (NO_2_^−^) and nitrite (NO_3_^−^) production was measured as a surrogate for NO production using a commercially available Nitrate/Nitrite colorimetric assay kit (Cayman Chemical, Ann Arbor, MI, USA) as previously described.^8^ Values were normalized to protein concentration of the samples measured using the Bradford assay as previously described.^7^

### Detection of reactive oxygen species and reactive nitrogen species

A DCFH-DiOxyQ fluorogenic probe based kit (OxiSelect, Cell Biolab, San Diego, CA, USA) was used to assess reactive oxygen species (ROS) and reactive nitrogen species (RNS) according to the manufacturers instructions. This kit measured the oxidation of 7'-dichlorodihydrofluorescin (DCFH) to highly fluorescent 2',7'-dichlorodihydrofluorescein (DCF) molecule.

Relative fluorescence was measured using a plate reader (Molecular Devices, Silicon Valley, CA, USA) at excitation and emission wavelengths of 480 and 530 nom, respectively. Fluorescence data were converted into concentrations based on dichlorodihydrofluorescin standard curves and normalized to protein concentration of the samples.

### Western blotting

Antibodies for p-eNOS^Ser1177^, total eNOS, p-Akt^Ser473^, total Akt, p-ERK^Thr 202/204^, total ERK and actin were purchased from Cell Signal Technology (Beverly, MA, USA). Cell homogenates were prepared and western blots were conducted as previously described using 40 μg protein per well.^8^ Blots were done in triplicate and band densities were quantified with a Kodak Gel Logic 1500 Imaging System and Kodak Molecular Imaging Software (Kodak MI v 4.0, Eastman Kodak Company, New Haven, CT, USA).

### Statistical analyses

Responses to insulin for each treatment condition were compared using an unpaired *t*-test for eNOS, Akt, ERK and NO experiments (SPSS V.21). For ROS experiments, data were analyzed using one-way ANOVA (SPSS V.21) and when main effects were detected, Tukey’s *post hoc* tests was conducted to determine differences between groups. *P*<0.05 was considered significant. Data are expressed as mean±s.e. of four to six independent experiments.

## Results

**Figure 1:**
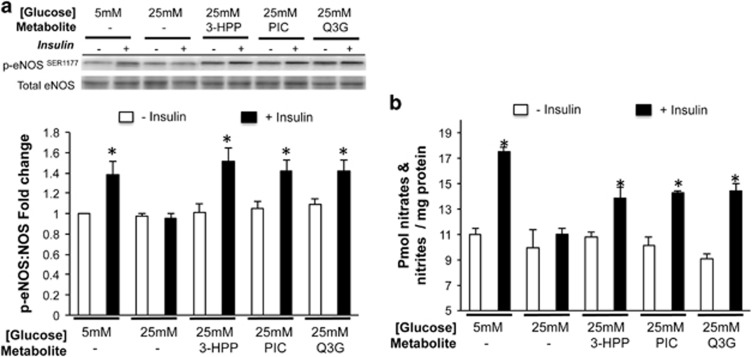
eNOS phosphorylation at Serine 1177 (**a**) and NO production (**b**) in human aortic endothelial cells grown in control (5 mM) or high (25 mM) glucose. After 48 h of incubation in control conditions NO production and eNOS phosphorylation were increased following insulin stimulation (10 min, 100 nM). However, 48 h incubation in high glucose conditions abolished insulin stimulated increases in NO and eNOS phosphorylation. Pre-treatment for 24 h with either Q3G, 3-HPP or PIC prior to 48 h incubation in high glucose preserved insulin-stimulated NO production and eNOS phosphorylation. Data are presented as mean±s.e. of four to six independent experiments. *P<0.05.

**Figure 2:**
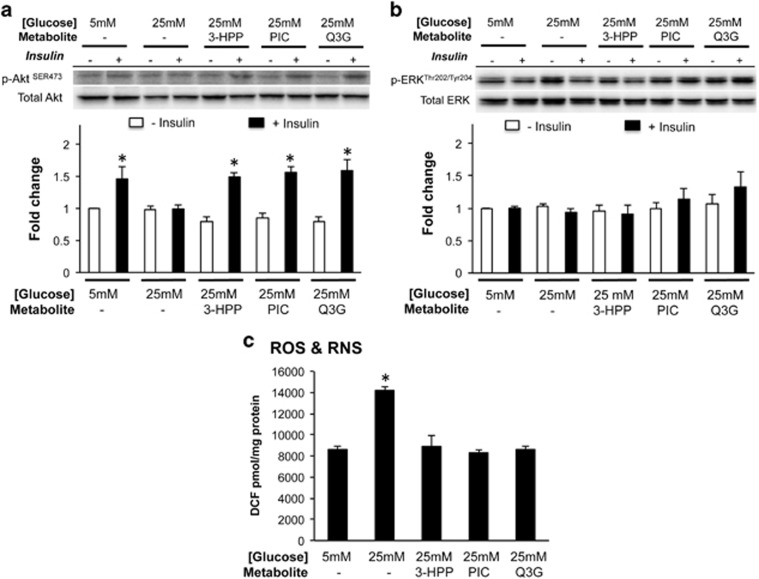
Akt phosphorylation at Serine 473 (**a**), ERK phosphorylation at Threonine 202 and Tyrosine 204 (**b**) and ROS & RNS (**c**) in human aortic endothelial cells grown in control (5 mM) or high (25 mM) glucose. After 48 h of incubation in control conditions Akt phosphorylation was increased following insulin stimulation (10 min, 100 nM). However, 48 h incubation in high glucose conditions abolished insulin-stimulated Akt phosphorylation and ROS/RNS levels were markedly greater. ERK phosphorylation was similar among conditions. Pre-treatment for 24 h with either Q3G, 3-HPP or PIC prior to 48 h incubation in high glucose preserved insulin-stimulated Akt phosphorylation and prevented the rise in ROS & RNS as determined by measuring DCF concentrations. Data are presented as mean±s.e. of four to six independent experiments. *P<0.05.

Basal NO production and p-eNOS^Ser1177^: eNOS were similar between HAECs grown in control (5 mM) or high (25 mM) glucose conditions (Figure 1). Insulin stimulation markedly increased both NO production (Figure 1) and p-eNOS^Ser1177^: eNOS in HAECs treated with 5 mM, but not 25 mM glucose. However, individual pre-incubation with Q3G, or 3-HPP, or PIC preserved both insulin-stimulated NO production and eNOS^Ser1177^ phosphorylation under high glucose conditions (Figure 1). eNOS activity can be modulated by phosphorylation by Akt or ERK, resulting in an increase or decrease in eNOS activity and NO production, respectively. As expected, high glucose conditions suppressed insulin-stimulated Akt phosphorylation vs. controls, whereas as pre-treatment with flavonoid metabolites preserved Akt phosphorylation (Figure 2). ERK phosphorylation was unchanged regardless of insulin stimulation (Figure 2). HAECs grown in high glucose conditions experienced a marked increase in ROS/RNS, that was prevented by pre-incubation with each of the flavonoid metabolites tested (Figure 2).

## Discussion

Acute and chronic hyperglycemia are associated with endothelial dysfunction.^15^ We used an *in vitro* model of hyperglycemia consisting of human aortic endothelial cells grown in high glucose (25 mM) conditions, that corresponds to blood levels of ~450 mg dl^−1^. Such concentrations may be encountered in patients with poorly controlled Type 1 or 2 diabetes. We present evidence that three metabolites of dietary flavonoids, that is, Q3G, 3-HPP, and PIC, preserve insulin-stimulated signaling to Akt and eNOS to an extent that maintains NO generation during high glucose conditions. These vascular benefits might be secondary to the antioxidant properties of flavonoid metabolites. For instance, all metabolites prevented the increase in ROS/RNS production otherwise evoked by high glucose. While the precise mechanisms were not explored in this study, increased ROS have potential to compromise NO availability via mechanisms including: (i) the formation of peroxynitrite by superoxide combining with NO; (ii) compromised eNOS enzyme expression/activity and/or (iii) the oxidation of BH_4_ to BH_2_ resulting in eNOS uncoupling.^16^

There is emerging evidence that flavonoid metabolites may independently have bioactivity that improves vascular function. For example, Najmanova *et al.* report that 3-HPP evokes relaxation of rat aortic rings but the mechanism was not identified.^17^ Data from our experiments demonstrate that 3-HPP preserves insulin-stimulated eNOS phosphorylation and NO production during high glucose conditions, which may be broadly interpreted to provide a potential mechanism underlying the observations reported by Najmanova *et al.* Others have reported that Q3G prevents insulin resistance in endothelial cells evoked by acute (30 min) palmitate incubation,^18^ and that PIC augments NO release in endothelial cells challenged with 100 μM palmitate for 12 h.^19^ Our findings that Q3G and PIC preserve insulin-stimulated Akt and eNOS phosphorylation, and nitrate/nitrite generation are congruent with these observations, and extend them to the context of glucotoxicity.

Earlier we demonstrated that quercetin lowers blood pressure in hypertensive animals and humans that concurrently exhibit characteristics of metabolic syndrome.^4, 20^ Further, when humans with metabolic syndrome are treated with a mixture of quercetin, green tea extract, grape seed extract, and resveratrol similar benefits concerning arterial blood pressure are realized.^8^ Data from the current study provide proof of concept that the antihypertensive effects exerted by quercetin and/or the flavonoid mixture we reported previously may result, at least in part, from the bioactivity of flavonoid metabolites, and not only the parent compounds.
